# Metformin Delays Satellite Cell Activation and Maintains Quiescence

**DOI:** 10.1155/2019/5980465

**Published:** 2019-04-24

**Authors:** Theodora Pavlidou, Milica Marinkovic, Marco Rosina, Claudia Fuoco, Simone Vumbaca, Cesare Gargioli, Luisa Castagnoli, Gianni Cesareni

**Affiliations:** ^1^Department of Biology, Tor Vergata University, 00133 Rome, Italy; ^2^IRCCS, Fondazione Santa Lucia, Rome, Italy

## Abstract

The regeneration of the muscle tissue relies on the capacity of the satellite stem cell (SC) population to exit quiescence, divide asymmetrically, proliferate, and differentiate. In age-related muscle atrophy (sarcopenia) and several dystrophies, regeneration cannot compensate for the loss of muscle tissue. These disorders are associated with the depletion of the satellite cell pool or with the loss of satellite cell functionality. Recently, the establishment and maintenance of quiescence in satellite cells have been linked to their metabolic state. In this work, we aimed to modulate metabolism in order to preserve the satellite cell pool. We made use of metformin, a calorie restriction mimicking drug, to ask whether metformin has an effect on quiescence, proliferation, and differentiation of satellite cells. We report that satellite cells, when treated with metformin *in vitro*, ex vivo, *or in vivo*, delay activation, Pax7 downregulation, and terminal myogenic differentiation. We correlate the metformin-induced delay in satellite cell activation with the inhibition of the ribosome protein RPS6, one of the downstream effectors of the mTOR pathway. Moreover, *in vivo* administration of metformin induces a belated regeneration of cardiotoxin- (CTX-) damaged skeletal muscle. Interestingly, satellite cells treated with metformin immediately after isolation are smaller in size and exhibit reduced pyronin Y levels, which suggests that metformin-treated satellite cells are transcriptionally less active. Thus, our study suggests that metformin delays satellite cell activation and differentiation by favoring a quiescent, low metabolic state.

## 1. Introduction

Skeletal muscle regeneration relies on the dynamic interplay between satellite cells (SCs) and their environment, the stem cell niche [1]. In the adult muscle, under resting conditions, SCs are mitotically quiescent [2]. Following damage, they are activated and divide asymmetrically. One daughter cell returns to quiescence to reconstitute the stamina pool while the other one proliferates and differentiates to eventually form new myofibers [3]. In healthy conditions, SCs are only sporadically activated to counterbalance physiological tissue turnover. As a consequence, muscle mass loss is prevented. The stability of the SC pool and the integrity of the stem cell niche, however, are affected by aging or disease [4]. In pathological conditions, as in Duchenne muscular dystrophy (DMD), chronic inflammation stimulates SC proliferation and differentiation by sending sustained regeneration signals. This phenomenon contributes to the exhaustion of the SC pool and the ensuing decrease in the regeneration potential [5].

Metabolic flexibility controls the balance between stem cell fates, as unique bioenergetic demands underlie quiescence, stem cell proliferation, and lineage specification [6]. In several tissues, cellular quiescence is associated with low metabolic activity, little mitochondrial respiration, reduced translational rates, and activation of autophagy in order to provide nutrients for survival [7]. On the other hand, stem cell activation, including satellite cell activation, is characterized by elevated energy demands and is mediated by an oxidative respiration to glycolytic metabolism shift [8, 9]. Specifically, it has been demonstrated that different metabolic pathways take part in the establishment of the quiescent state. SIRT1, a nutrient sensor, regulates the autophagic flux in SC progeny, and loss of SIRT1 leads to a delay in SC activation [10]. Rodgers et al. reported that mTOR activity, a known inhibitor of autophagy, is necessary for the transition of SCs and fibroadipogenic progenitors (FAP) from a G_0_ phase to a G_Alert_ quiescent phase, where G_Alert_ stem cells have higher propensity to cycle, increased mitochondrial activity, and enhanced differentiation kinetics [11]. Furthermore, we have recently shown that AMPK activation by the antidiabetic drug metformin plays a negative role in C2C12 skeletal muscle differentiation and prevents permanent exit from the cell cycle, mimicking in this way the quiescent “reserve cell” phenotype [12].

Thus, perturbing stem cell metabolism may influence muscle regeneration. Cerletti et al. have demonstrated that short-term calorie restriction increases the percentage and the myogenic function of Pax-7-expressing cells in the muscles of young and old mice [13]. They associated this phenotype with an increased mitochondrial number in SCs derived from mice fed with a low-calorie diet and an enhanced transplantation potential. In accordance, chronic treatment with metabolic remodeling agents, such as AICAR and PPAR*δ* agonist, favors oxidative metabolism in mdx mice [14, 15]. The natural phenol resveratrol is also reported to induce oxidative metabolism in mdx mice by increasing the levels and activity of SIRT1 [16] while favoring utrophin gene expression [17]. In order to further investigate the role of metabolic reprogramming in skeletal muscle stem cell fate, here we examine the effect of metformin on SC activation and differentiation *in vivo* and ex vivo.

Metformin (1,1-dimethylbiguanide hydrochloride) is a calorie restriction mimicking drug that is widely prescribed for the treatment of hyperglycemia in individuals with type II diabetes [18]. The main targets of metformin are hepatocyte mitochondria where it disrupts respiratory chain complex I leading to a decrease in the ATP/AMP ratio [19]. As a result of AMP accumulation, AMP-activated protein kinase (AMPK) is activated [20]. AMPK is a serine/threonine kinase which works as a sensor of changes in cellular energy levels and metabolic stress [21]. Different studies have described beneficial effects of metformin on the prevention and treatment of cancer as it exerts an antiproliferative effect by inhibiting mTOR. Specifically in MCF7 breast cancer cells, it has been shown that metformin inhibits cell growth through negative regulation of the mTOR pathway and 30% reduction in global protein synthesis [22]. The molecular mechanism underlying this antiproliferative effect has been characterized by revealing the proteomic profile of metformin-treated MCF7 cells [23].

In skeletal muscles, metformin has been demonstrated to protect mouse muscles from cardiotoxin-induced damage [24] and ameliorate the PGC1A and utrophin expression in dystrophic mice [25]. Notably, metformin is now tested in clinical trials for the improvement of muscle function in patients with Duchenne muscular dystrophy [26, 27].

Here, we focus on the effect of the drug on satellite cell activation and differentiation *in vivo* and ex vivo. Our results indicate that metformin delays the activation, Pax7 downregulation, and terminal myogenic differentiation of SCs. This belated SC activation is paralleled by a delayed regeneration of skeletal muscle injury, inhibition of mTOR, and reduced RPS6 phosphorylation that induce the low metabolic state associated with quiescence.

## 2. Materials and Methods

### 2.1. Animal Procedures

An equal number of 1.5-month-old C57BL/6 mice was used for control (*n* = 12) and metformin-treated (*n* = 12) experimental groups. Twelve C57BL/6 mice were pretreated with 300 mg/kg body weight of metformin (Sigma-Aldrich PHR1084) diluted in water for 21 days. Muscle crush injury was induced by cardiotoxin in already-anesthetized mice. Anesthesia was induced by an intramuscular injection of physiologic saline (10 ml/kg) containing ketamine (5 mg/ml) and xylazine (1 mg/ml). 10 *μλ* of cardiotoxin isolated from *Naja pallida* (Latoxan L81-02) was intramuscularly administered into the tibialis anterior (TA), quadriceps, and gastrocnemius (GC) muscle, and after 4 and 7 days of treatment, control and metformin-treated mice were sacrificed. Metformin administration was maintained during the whole regeneration period. Isolated tibialis anterior (TA) muscles were snap frozen in OCT for cryosectioning with a Leica cryostat while the rest of the hind limb muscles were homogenized for cell isolation. Experiments on animals followed the rules of good animal experimentation of I.A.C.U.C. and obtained ethical approval released on 16/09/2011 from Italian Ministry of Health (protocol #163/2011-B).

### 2.2. SC Isolation

Hind limb muscles were gently isolated from mice, nonmuscle tissue was removed, and muscles were minced and subjected to enzymatic dissociation for 45 min at 37°C. The enzymatic mix was composed of 2 *μ*g/ml collagenase A (Roche cat#10103586001), 2.4 U/ml dispase II (Roche cat#04942078001), and 0.01 mg/ml DNase I (Roche cat#04716728001) diluted in D-PBS with calcium (130 mg/l) and magnesium (200 mg/l). Enzymatic dissociation was stopped by the addition of Hank's balanced salt solution (HBSS), and the cell suspension was filtered progressively through a 100, 70, 40, and 30 *μ*m cell strainer. Cells were incubated with the appropriate antibodies conjugated with magnetic microbeads and isolated using the MACS separation technology. Lineage negativity characterization was performed by using the antibodies CD45 (Miltenyi cat#130-052-301) and CD31 (Miltenyi cat#130-097-418). The CD45^−^/CD31^−^cells were further positively selected with an a7-integrin microbead antibody (Miltenyi cat#130-104-261). SCs were selected as CD45^−^/CD31^−^/a7-integrin^+^ cells, and cell purity was confirmed by Pax7 expression.

### 2.3. Single-Fiber Isolation

To isolate single myofibers, EDL muscles were digested with 0.2 *μ*g/*μ*l collagenase A (Roche cat#10103586001) in Tyrode's medium (Sigma cat#T2145-10X1L) for 1 h at 37°C with gently shaking every 10 min. Muscles were dissociated by gentle triturating and were washed several times to eliminate cellular debris and contaminating cells. EDL myofibers were then cultured in Tyrode's medium supplemented with 10% heat-inactivated fetal bovine serum and penicillin-streptomycin/antimycotic (100 U/100 g/ml).

### 2.4. Cell Cultures

SCs were cultured in a gelatin-coated dish with Dulbecco's modified Eagle's medium (DMEM) supplemented with 20% heat-inactivated fetal bovine serum, 10% heat-inactivated horse serum, 2% chicken embryo extract, penicillin-streptomycin (100 U/100 g/ml), 1 mM sodium pyruvate, and 10 mM HEPES. SCs spontaneously differentiate into multinucleated myotubes in culture medium. For cell proliferation and western blot experiments, SCs were expanded for four days in Cytogrow medium (Resnova) to allow a sufficient number of cells. SCs were then trypsinized and plated in culture medium. SCs were treated with 2 mM metformin every 48 h. The control samples were treated with an equal quantity of PBS.

### 2.5. Histological Analysis

Evaluation of the percentage of centronucleated myofibers was carried out by H&E staining. Centronucleated fibers were counted in 20 *μ*m thick histological sections of skeletal muscle tissues, and a total number of at least 3000 fibers of the damaged area were counted for each mouse. The results were expressed as the percentage of centronucleated fibers to the total fiber number of the damaged area. Analysis was performed using ImageJ software.

### 2.6. Immunofluorescence

Fixation of cryosections was performed by incubation with 4% of paraformaldehyde (PFA) for 5 minutes, followed by rinsing with PBS containing 1% BSA and 0.1% Triton X-100 (5 min at RT), blocking with PBS containing 0.2% Triton X-100 and 20% goat serum (2 h at RT), and incubating with the primary antibody overnight at 4°C. Samples were then washed three times and incubated with the secondary antibody for 1 h at RT. Next, cryosections were washed three times and incubated with Hoechst (1 mg/ml, 5 min at RT), washed again, and mounted. SCs and single myofibers were fixed with 2% paraformaldehyde (PFA) for 15 minutes and permeabilized in 0.1% Triton X-100 for 5 min. Blocking was performed with 1% PBS containing 10% serum and 0.1% TritonX-100 for 1 h at RT. The cells were stained with the primary antibody for 1 h at RT, washed three times with PBS, and incubated with the secondary antibody for 30 min at RT.

Single myofibers were blocked with PBS containing 0.2% Triton X-100 and 20% goat serum for 2 h at RT and incubated with the primary antibody overnight. Single myofibers were washed three times and incubated with the secondary antibody for 1 h at RT. The fibers were finally washed three times, and nuclei were counterstained with Hoechst 33258 (1 mg/ml, 5 min at RT). The antibodies used were the following: rabbit anti-MyoD (1 : 20, Santa Cruz sc-760), mouse anti-MyHC (1 : 2, DSHB), rabbit anti-laminin (1 : 200, Sigma cat#L9393-.2ML), mouse anti-Pax7 (1 : 15, DSHB), mouse anti-myogenin (eBioscience cat#14-5643), rabbit anti-phospho RPS6 (Cell Signaling cat#3985), anti-rabbit secondary antibody conjugated with Alexa Fluor 555 (1 : 100, Life Technologies A-21428), and anti-mouse secondary antibody conjugated with Alexa Fluor 488 (1 : 100, Life Technologies A-11001).

### 2.7. Cell Proliferation Assay by BrdU/EdU

Cell proliferation was measured by BrdU (GE Healthcare) or EdU (Life Technologies Inc.). BrdU and EdU (5-ethynyl-2′-deoxyuridine) are nucleoside analogs of thymidine that are incorporated into DNA during active DNA synthesis. The cells were plated at the desired density and treated for 24 h with a 10 *μ*M EdU solution prepared with culture media. The cells were fixed and permeabilized according to the manufacturer's protocol using 3.7% formaldehyde in PBS, followed by a 0.5% Triton® X-100 permeabilization step. After that, cells were incubated with the Click-iT EdU reaction mix (1X Click-iT® reaction buffer CuSO, Alexa Fluor® azide, and reaction buffer additive) for 30 min at room temperature protected from light. Finally, the samples were washed twice with 3% BSA in PBS and stained for nuclei. Images were acquired with a Leica fluorescent microscope (DMI6000B).

### 2.8. Cell Growth Curve

Proliferation of *in vitro* metformin-treated and control SCs was monitored by counting the total cell nuclei per image field after 2 and 4 days of treatment. The monitoring of SC cell growth was performed according to our previous report [12]. Briefly, the cells were plated in the same initial number in all conditions, and at each time point, cells were fixed with 2% paraformaldehyde solution in 1X PBS for 10 minutes at room temperature (RT). Following staining with 2 *μ*g/ml Hoechst 33342 (Thermo Fisher Scientific) in 0.1% Triton X-100 (*v*/*v*) in 1X PBS for 5 minutes, acquisition of images was carried out by a Leica DM6000B (Leica Microsystems) automated fluorescence microscope. In total, 25 fields/wells were acquired by a 5 × 5 matrix covering the whole surface of the sample. Counting of the nuclei was performed by using the CellProfiler software, and the data were represented as mean of four independent cell isolations and biological replicates. Doubling time analysis was performed by using the nonlinear regression/exponential growth equation tool in GraphPad Prism. All replicates were analyzed separately. Doubling time is reported as hours ± SEM.

### 2.9. Immunoblotting

Protein extraction and western blot analysis were performed as reported from our previous publication [12]. The antibodies used were as follows: mouse anti-Pax7 (1 : 500, DSHB AB_528428), rabbit anti-MyoD (1 : 500, Santa Cruz sc-760), mouse anti-MyHC (1 : 500, DSHB MF20), rabbit anti-Tom20 (1 : 1000, Cell Signaling 42406), mouse anti-Sirt1 (1 : 1000, Cell Signaling 8469), rabbit anti-acH3 (1 : 1000, Cell Signaling 9649), rabbit anti-phospho AMPK (Thr172) antibody (1 : 1000, Cell Signaling 2535), rabbit anti-AMPK (1 : 1000, Cell Signaling 2603), rabbit anti-phospho P70S6K (Thr421/Ser424) (1 : 1000, Cell Signaling 9204), rabbit anti-P70S6K (1 : 1000, Cell Signaling 9202), rabbit anti-phospho RPS6 (Ser240/244) antibody (1 : 1000, Cell Signaling 2215), rabbit anti-RPS6 (1 : 1000, Cell Signaling 2217), and rabbit anti-tubulin antibody (1 : 500, Santa Cruz sc-9104). Densitometric analysis was performed using ImageQuant. Normalization of phosphorylated and total proteins was performed by using tubulin or vinculin. Finally, the ratio between the phosphorylated and total protein was indicated.

### 2.10. Mito Stress Analysis

Cells were plated at a density of 3000 cells/well on Seahorse XF96 Cell Culture Microplates (Agilent) in Cytogrow medium overnight.

Cells were treated in SC culture medium with 2 mM metformin or PBS as a control for 24 h.

The Mito Stress Test was performed according to Agilent's recommendations, stimulating the mitochondrial respiratory chain with 1 *μ*M oligomycin, 1.5 *μ*M FCCP, and 1 *μ*M rotenone/antimycin.

For normalization, immediately after the assay completion, cells were fixed with 2% PFA for 20 minutes at RT and washed 3 times with 1X PBS. Nuclei were stained with Hoechst 33342 (1 : 5000) in 0.1% Triton X-100 for 5 minutes at RT and washed 3 times with 1X PBS. The central 10x field of each well was acquired, and counting of nuclei was performed with the CellProfiler software. The total number of nuclei per well was estimated calculating the field-to-well ratio. OCR values were reported as pmol O_2_/min/1000 cells.

### 2.11. Apoptosis Detection

For apoptosis detection by annexin V/PI, cells were analyzed by flow cytometry after staining with annexin V/PI following protocol instructions (Cell Signaling Technology). After treatment, adherent and floating cells were collected, washed twice with ice-cold PBS, and suspended in annexin V binding buffer. 1 *μ*l of annexin V-FITC conjugate and 12.5 *μ*l of PI were added to each sample, and samples were incubated for 10 minutes on ice protected from light. Stained cells were diluted in ice-cold buffer and directly analyzed by a BD FACSCalibur flow cytometer. This double staining allows highlighting four distinct cell populations: alive cells (annexin V negative, PI negative), early apoptotic cells (annexin V positive, PI negative), late apoptotic cells (annexin V positive, PI positive), and necrotic cells (annexin V negative, PI positive). Cell percentage for each population was determined using the FlowJo software (FlowJo, LLC, USA).

### 2.12. RNA Level Detection by Pyronin Y Staining

Cells were resuspended in Hank's solution, washed two times, and fixed in cold methanol : acetone (4 : 1) for 30 min at 4°C. The samples were then washed twice and incubated with 0.5 *μ*g/ml pyronin Y for 30 min at 37°C. Before analysis by the BD FACSCalibur flow cytometer, cells were transferred into ice for at least 10 min. Pyronin Y is excited at 488 nm, emits at 575 nm, and analyzed in a linear scale.

### 2.13. Statistical Analysis

All the data presented are mean values ± SEM of at least three experiments. Student's *t*-test or ANOVA statistical analysis was used to estimate the significance of the observed differences in the means in all experiments. The differences were considered significant at *p* < 0.05.

## 3. Results

### 3.1. Metformin Delays Pax7 Downregulation

**Figure 1:**
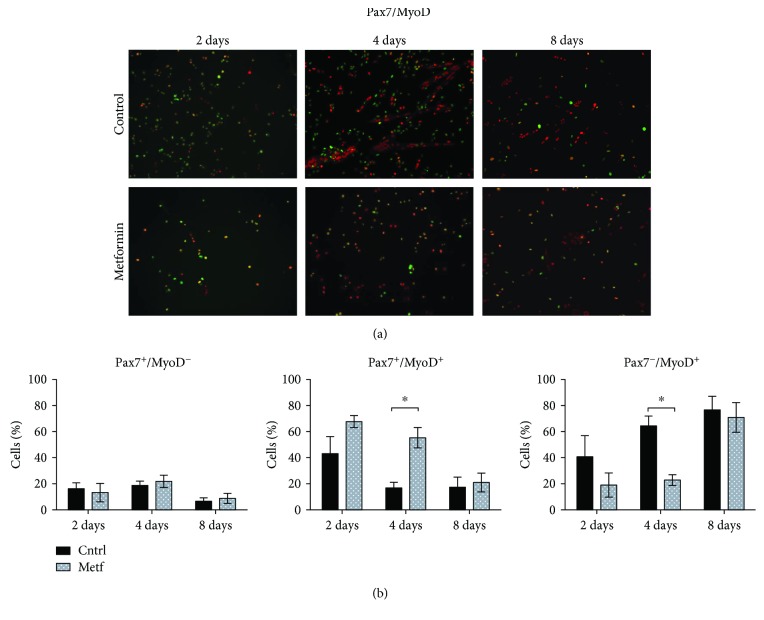
Metformin delays Pax7 downregulation. (a) SCs were isolated from C57BL/6 mice, attached onto plastic plates, and treated with 2 mM metformin for 2, 4, and 8 days. SCs were further analyzed by immunofluorescence microscopy for the expression of Pax7 and MyoD. (b) The percentage of cells expressing Pax7^+^/MyoD^−^, Pax7^+^/MyoD^+^, and Pax7^−^/MyoD^+^ after 2, 4, and 8 days of treatment with metformin was calculated in three independent cell isolations and experimental replicates (*n* = 3). Statistical significance was evaluated by the ANOVA test (^∗^ *p* < 0.05).

Quiescent SCs express the transcription factor Pax7 [28]. After activation, each SC divides asymmetrically, producing one myoblast Pax7^+^, MyoD^+^, that amplifies and differentiates and one quiescent stem cell. When myoblasts commit to skeletal muscle differentiation, the expression of Pax7 is downregulated and they stop proliferating and express the myogenic factor myogenin [3]. In order to understand the role of metformin in SC proliferation, we isolated SCs from C57BL/6 mice by microbead technology as CD45^−^, CD31^−^, and a7-integrin^+^ cells and treated them with 2 mM metformin for 2, 4, and 8 days in DMEM-supplemented medium. Four days after plating, most SCs already express MyoD irrespective of metformin treatment. We observed, however, that the downregulation in the expression of Pax7 is delayed in metformin-treated SCs (Figures 1(a) and 1(b)). The percentage of SCs that remains positive for both Pax7 and MyoD is significantly higher after 4 days of metformin treatment when compared to the control, while the fraction of SCs committed to myogenic differentiation (Pax7^−^/MyoD^+^) remains significantly lower in the treated sample. After 8 days, the fraction of cells that do not express Pax7 (Pax7^−^/MyoD^+^) is similar in both the metformin-treated and untreated cultures.

We further monitored the expression of Pax7 and MyoD by western blot analysis. This analysis, however, (Figure S1) did not reveal significant differences in the kinetic of expression of the two myogenic markers suggesting that the differences observed at the level of single-cell analysis are blurred in the bulk analysis.

**Figure 2:**
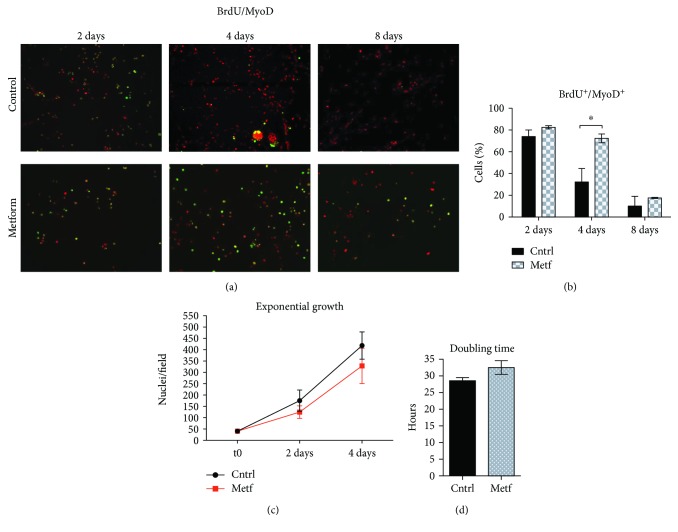
Metformin delays SC cycle exit. (a) SCs were treated upon attachment for 2, 4, and 8 days with 2 mM metformin. 24 h before fixation, a BrdU labeling reagent was added to the culture medium and cells were further analyzed by fluorescence microscopy for the incorporation of BrdU and the expression of MyoD. (b) The percentage of cells expressing BrdU^+^/MyoD^+^ after 2, 4, and 8 days of treatment with metformin was calculated after three independent cell isolations and experimental replicates (*n* = 3). Statistical significance was evaluated by the ANOVA test (^∗^ *p* < 0.05). (c) Growth curve of control and metformin-treated SCs. SCs were treated with 2 mM metformin *in vitro* for 2 and 4 days, and the number of nuclei per field was counted by immunofluorescence microscopy. The initial number of plated cells was the same in each condition. The growth curves are derived from four independent biological replicates (*n* = 4). Statistical significance was evaluated by the ANOVA test (^∗^ *p* < 0.05). (d) Doubling time analysis of control and metformin-treated cells. The analysis was performed using the nonlinear regression/exponential growth equation tool in GraphPad Prism. The bar graph represents the average of four independent biological replicates (*n* = 4). Statistical significance was evaluated by Student's *t*-test (^∗^ *p* < 0.05).

We also investigated SC proliferation by monitoring the number of SCs that incorporate BrdU and noticed that it is significantly higher in the metformin-treated sample at day 4 of treatment (Figures 2(a) and 2(b)), a result that is in accordance with the belated Pax7 downregulation. In spite of the higher number of metformin-treated cells still actively incorporating BrdU at day 4, the total number of cells is lower in the treated sample than in control as indicated by the exponential growth curve (Figure 2(c)). In addition, by calculating the doubling time, we observed that metformin-treated SCs are characterized by a longer doubling time compared to the control. These differences, however, are not statistically significant (Figure 2(d)). These observations are consistent with the conclusion that metformin holds the SCs for a longer time in a predifferentiation stage where they express both Pax7 and MyoD and are still actively replicating.

### 3.2. Metformin Delays SC Differentiation

**Figure 3:**
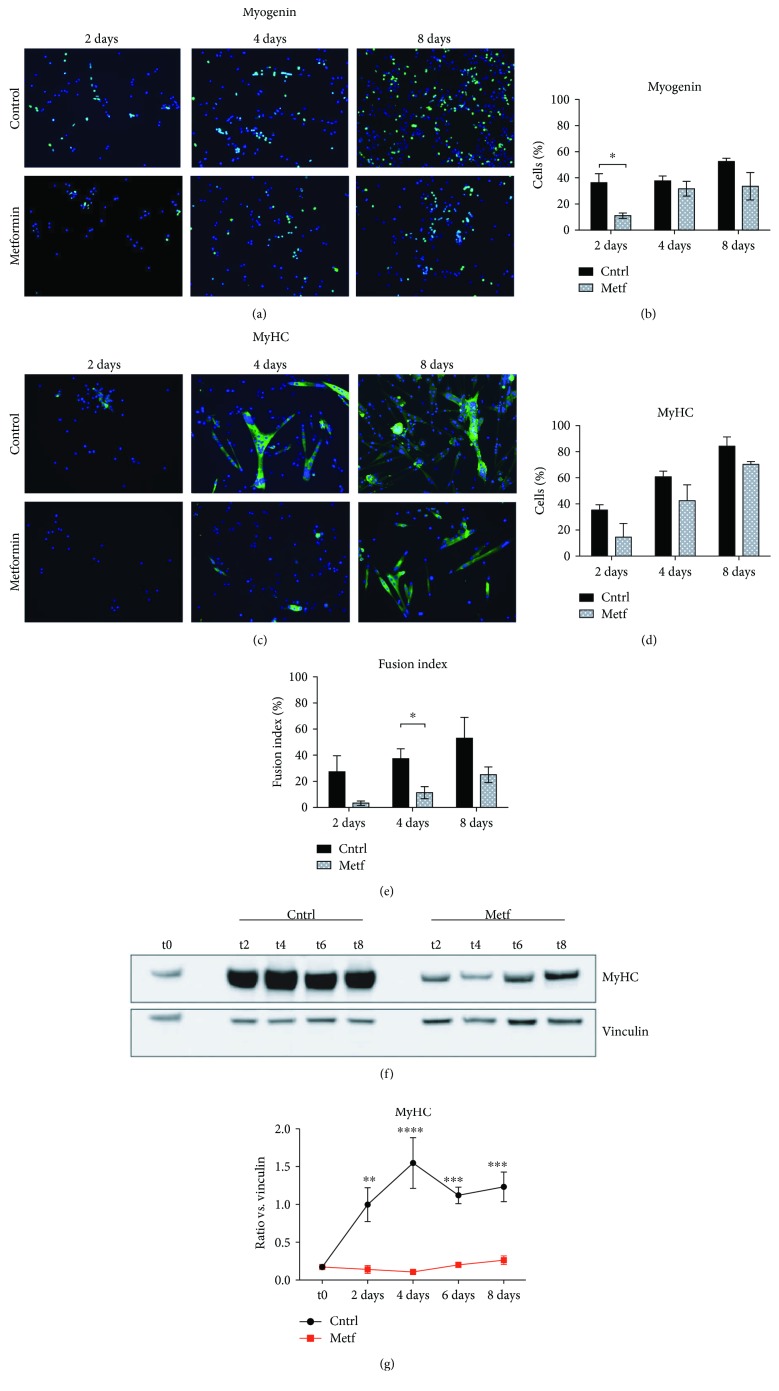
Metformin delays SC differentiation. (a) SCs were isolated from C57BL/6 mice, attached to a plastic dish, and treated with 2 mM metformin for 2, 4, and 8 days. The SCs were analyzed by immunofluorescence microscopy for the expression of the early myogenic marker myogenin. (b) The percentage of cells expressing myogenin after 2, 4, and 8 days of treatment with metformin was averaged from the results of three independent cell isolations and experimental replicates (*n* = 3). Statistical significance was evaluated by the ANOVA test (^∗^ *p* < 0.05). (c) SCs were treated upon attachment for 2, 4, and 8 days with 2 mM metformin and analyzed by fluorescence microscopy for the expression of the myosin heavy chain (MyHC). (d) The percentage of cells expressing MyHC after 2, 4, and 8 days of treatment with metformin was analyzed after three independent cell isolations and experimental replicates (*n* = 3). Statistical significance was evaluated by the ANOVA test (^∗^ *p* < 0.05). (e) The fusion index was calculated as the % of nuclei inside myotubes over the total number of *nuclei*. A myotube is defined as a cell expressing MyHC and containing at least three *nuclei* inside a continuous cell membrane. Statistical significance was evaluated by the ANOVA test (^∗^ *p* < 0.05) after three independent cell isolations and experimental replicates (*n* = 3). (f) Western blot analysis for the expression of MyHC of control and metformin-treated SCs after 2, 4, 6, and 8 days of treatment (t2, t4, t6, and t8, respectively). Vinculin was used as a loading control. (g) Quantitation graph of MyHC protein levels monitored by western blot in four independent cell isolations and biological replicates (*n* = 4). Statistical significance was evaluated by the ANOVA test (^∗∗∗∗^ *p* < 0.0001).

Given that metformin treatment delays the downregulation of Pax7 expression in SCs, we further asked whether metformin also affects SC terminal differentiation. As shown in Figure 3, metformin significantly defers the expression of the early differentiation marker myogenin after 2 days of treatment (Figures 3(a) and 3(b)), while it reduces the expression of myosin heavy chain (MyHC) (Figures 3(c) and 3(d)) and affects the formation of multinucleated myotubes (Figure 3(e)). The above results were confirmed by western blot analysis for the expression of MyHC protein levels in control and metformin-treated SCs. As shown in Figures 3(f) and 3(g), protein levels of MyHC are significantly lower in the metformin samples at all time points analyzed (2, 4, 6, and 8 days of treatment).

### 3.3. Metformin Delays the Activation and Proliferation of Myofiber-Associated SCs

**Figure 4:**
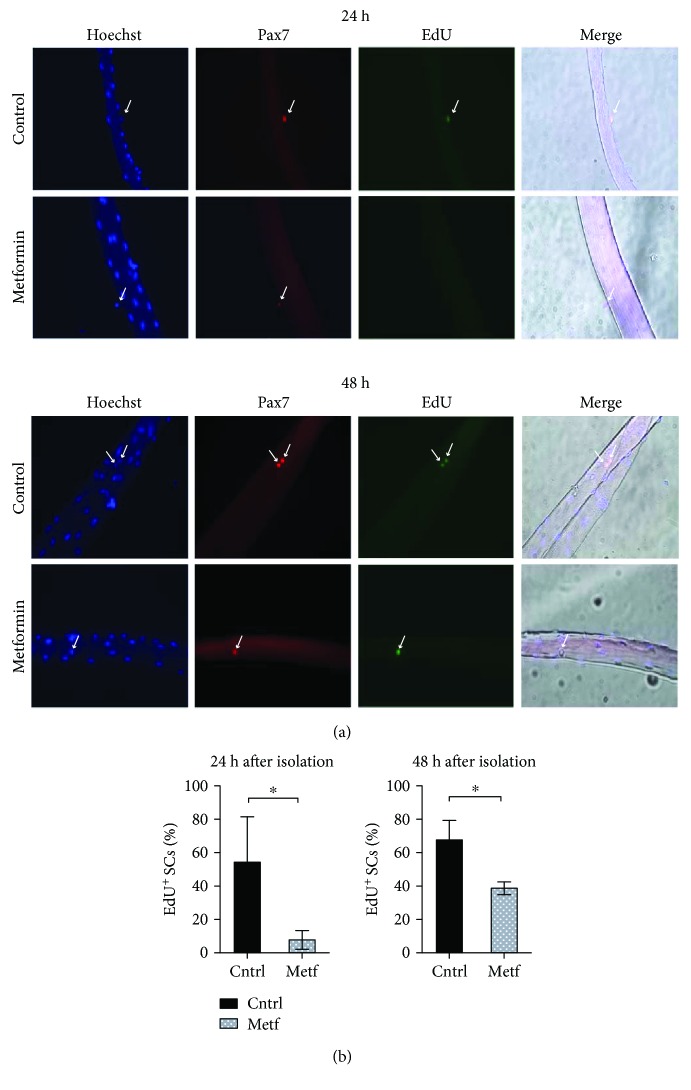
Metformin delays SC activation. (a) Myofibers were isolated from C57BL/6 mice and treated *in vitro* with 2 mM metformin in Tyrode's medium containing an EdU labeling agent. The SCs associated with the myofibers were analyzed by immunofluorescence microscopy for the expression of Pax7 and the incorporation of EdU after 24 h and 48 h of culture. (b) The percentage of SCs that have incorporated EdU after 24 h and 48 h in culture was calculated after three independent single-fiber isolations and experimental replicates. Statistical significance was evaluated by Student's *t*-test (^∗^ *p* < 0.05) (n 24 h control = 56 SCs, n 24 h metf = 54 SCs, n 48 h control = 68 SCs, and n 48 h metf = 52 SCs).

In order to study the effect of metformin on the transition from quiescence to the proliferative state, we isolated single myofibers from the extensor digitorum longus (EDL) muscle of C57BL/6 mice and treated them with 2 mM metformin for 24 h and 48 h. The propensity of the SC to cycle was evaluated by measuring the incorporation of EdU (5-ethynyl-2′-deoxyuridine), a thymidine analog (Figure 4). After 24 hours, fewer SCs incorporate EdU when compared to the control sample (Figure 4(b)). During the following 24 hours, the percentage of SCs incorporating EdU increases in the metformin sample while still remaining significantly lower compared to controls. These results suggest that metformin delays the transition of the fiber-associated SCs from quiescence to the active, proliferative state.

### 3.4. Metformin Downregulates RPS6 in Myofiber-Associated SCs

Activation of AMPK [20] and the ensuing inactivation of mTOR signaling [22] are two readouts of metformin treatment. Thus, we investigated the activation of the downstream mTOR effector RPS6 in SCs associated with single myofibers.

**Figure 5:**
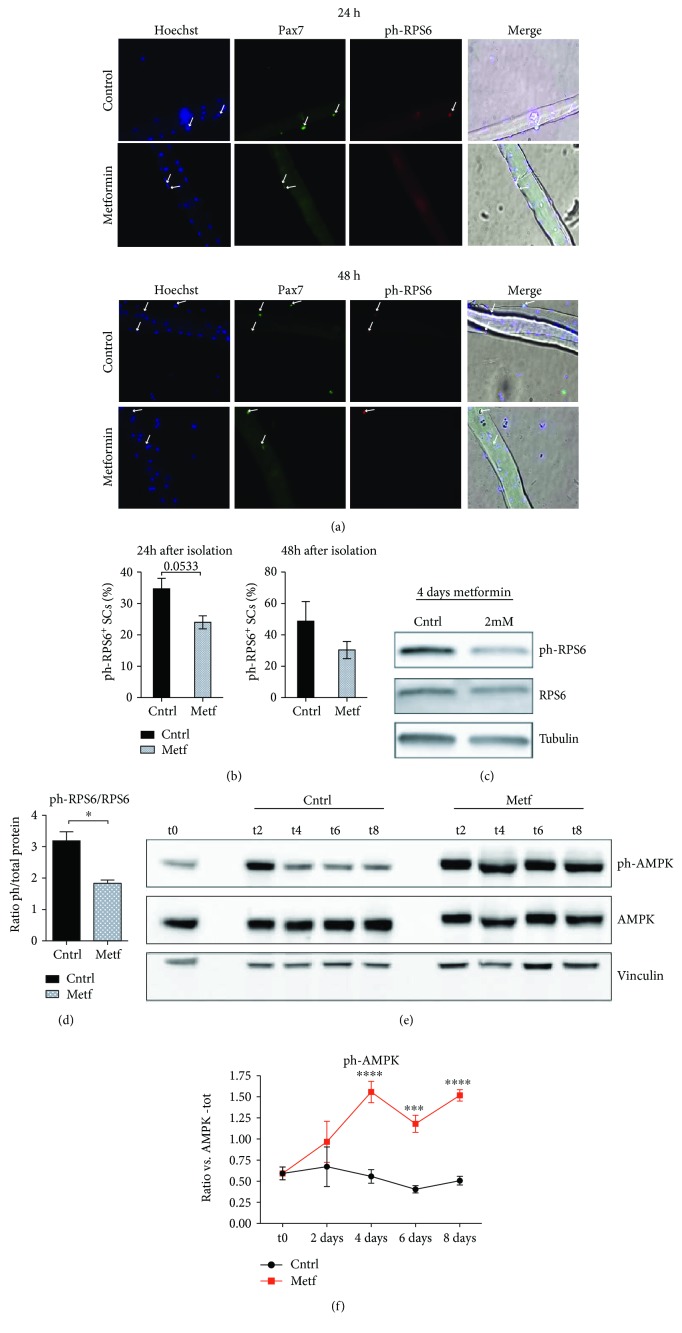
Metformin negatively modulates the phosphorylation of RPS6 in SCs. (a) Single myofibers were isolated from C57BL/6 mice and treated *in vitro* with 2 mM metformin. The SCs associated with the myofibers were analyzed by immunofluorescence microscopy for the expression of Pax7 and p-RPS6 after 24 h and 48 h of culture. (b) The percentage of SCs that are positive for p-RPS6 after 24 h and 48 h in culture was calculated after three independent single-fiber isolations and experimental replicates. Statistical significance was evaluated by Student's *t*-test (^∗^ *p* < 0.05) (number of counted SCs in each sample: 24 h control = 86, 24 h metf = 82, 48 h control = 72, and 48 h metf = 69). (c) Isolated SCs were treated *in vitro* for 4 days with 2 mM metformin, and protein extracts were analyzed by SDS-PAGE for the expression of ph-RPS6 and total RPS6 protein. Tubulin was used as a loading control. (d) Quantitation graph of ph-RPS6 to total RPS6 protein levels monitored by western blot in three independent cell isolations and biological replicates (*n* = 3). Statistical significance was evaluated by Student's *t*-test (^∗^ *p* < 0.05). (e) Western blot analysis for the expression of ph-AMPK and total AMPK protein of control and metformin-treated SCs after 2, 4, 6, and 8 days of treatment (t2, t4, t6, and t8, respectively). Vinculin was used as a loading control. (f) Quantitation graph of ratio ph-AMPK/total AMPK monitored by western blot in four independent cell isolations and biological replicates (*n* = 4). Statistical significance was evaluated by the ANOVA test (^∗∗∗∗^ *p* < 0.0001).

Isolated myofibers were treated *in vitro* with 2 mM metformin for 24 h and 48 h, and the phosphorylation of RPS6 was examined by immunofluorescence (Figure 5(a)). The levels of phosphorylated RPS6 (ph-RPS6) after 24 h and 48 h of treatment are lower in the metformin-treated myofibers compared to the untreated control (Figure 5(b)). The activation of RPS6 was also monitored in lysates of isolated SCs treated *in vitro* with 2 mM metformin for 4 days. After 4 days of metformin treatment, the fraction of RPS6 protein that is phosphorylated is lower (Figures 5(c) and 5(d)). The inhibition of RPS6 was accompanied by a significant activation of the AMPK, the main molecular target of metformin. As shown in Figures 5(e) and 5(f), the ratio of phosphorylated AMPK to total AMPK is significantly higher upon metformin treatment.

Given that RPS6 phosphorylation correlates with global protein synthesis, this result suggests that metformin negatively modulates protein synthesis of SCs when they are cultivated both *in vitro* after purification and in a condition in isolated myofibers that is more similar to their natural in vivo niche.

### 3.5. Metformin Delays Skeletal Muscle Regeneration *In Vivo*

**Figure 6:**
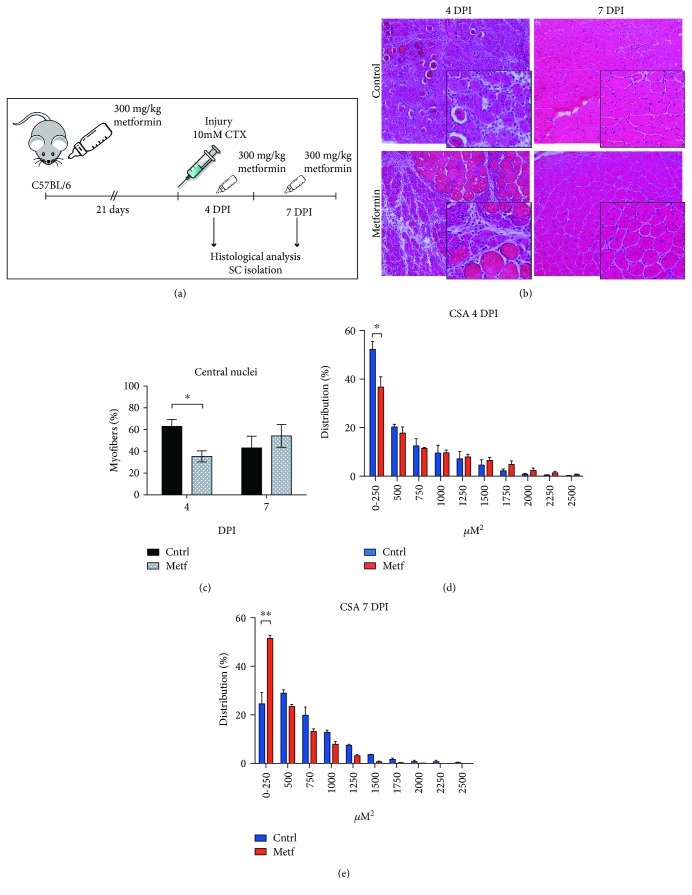
Metformin delays skeletal muscle regeneration *in vivo*. (a) Experimental design of the in vivo administration of metformin to cardiotoxin- (CTX-) injured C57BL/6 mice. (b) Representative images of H&E staining on TA muscle sections of control and metformin-treated mice at 4 and 7 days of post-cardiotoxin (CTX) injury (DPI). (c) Quantitation of the centrally located myonuclei was performed on TA muscle sections upon H&E staining of control and metformin samples. The percentage of centronucleated myofibers is reported as the total number of centronucleated myofibers to the total number of myofibers in the damaged muscle area. Statistical significance was evaluated by the ANOVA test (^∗^ *p* < 0.05) (n control = 3 mice, n metformin = 3 mice). (d) Cross-sectional area (CSA) distribution of myofibers in the control and metformin-treated mice 4 days after CTX injury (n control = 3 mice, (^∗^ *p* < 0.05). (e) Cross-sectional area (CSA) distribution of myofibers in the control and metformin-treated mice 7 days after CTX injury (n metformin = 3 mice, ^∗∗^ *p* < 0.01).

Next, we investigated the effect of metformin treatment on the activation of SCs *in vivo* during skeletal muscle damage and regeneration. C57BL/6 mice were pretreated with metformin for 21 days by administration in drinking water as shown in the experimental design (Figure 6(a)). Injection of cardiotoxin into the tibialis anterior (TA) was used to induce muscle damage. Regeneration was monitored at 4 and 7 days postinjury (DPI) while metformin treatment was maintained until the sacrifice of the animals. Newly formed myofibers can be readily distinguished in muscle cross sections for their small caliber and for the presence of centrally located myonuclei [1]. To evaluate the regeneration kinetics, we measured the number of centronucleated fibers in metformin-treated and untreated mice. As shown in Figures 6(b) and 6(c), the percentage of centrally nucleated myofibers at 4 DPI is significantly lower in the metformin-treated muscles compared to the control. On the contrary, at 7 DPI, the percentage of newly formed myofibers in the control sample decreases, while it increases in the metformin sample, even though this difference is not statistically significant (*p* value = 0.5030). This observation is compatible with the hypothesis that the muscles of the metformin-conditioned mice have a delay in the onset of the regeneration process and are still fully regenerating at 7 DPI. In agreement with this hypothesis, at 4 DPI, significantly smaller fibers (fiber group with size 0-250 *μ*Μ^2^) are observed in the muscles of the control mice (Figure 6(d)), while at 7 DPI, the number of myofibers with a smaller cross-sectional area is significantly higher in the metformin-treated regenerating muscles than in the control (Figure 6(e)), consistent with a delay in the completion of the regeneration process. In order to further investigate the role of metformin in skeletal muscle regeneration, we isolated SCs from the cardiotoxin-injured mice at days 1, 2, 4, and 7 postinjury and cultured them *in vitro*. Upon attachment, the expression of myogenic markers and terminal differentiation were monitored by immunofluorescence. SCs derived from mice that had received metformin exhibited delayed myotube formation *in vitro*, as shown by staining with myosin heavy chain (MyHC) antibodies. Control SCs differentiate readily *in vitro* already from the first day after injury, while the metformin-treated SCs achieve the same level of differentiation when they are purified from mice four days after injury (Figure S2). We conclude that metformin delays SC-dependent muscle regeneration.

### 3.6. The Activation and Proliferation of Myofiber-Associated SCs Are Delayed after *In Vivo* Administration of Metformin

**Figure 7:**
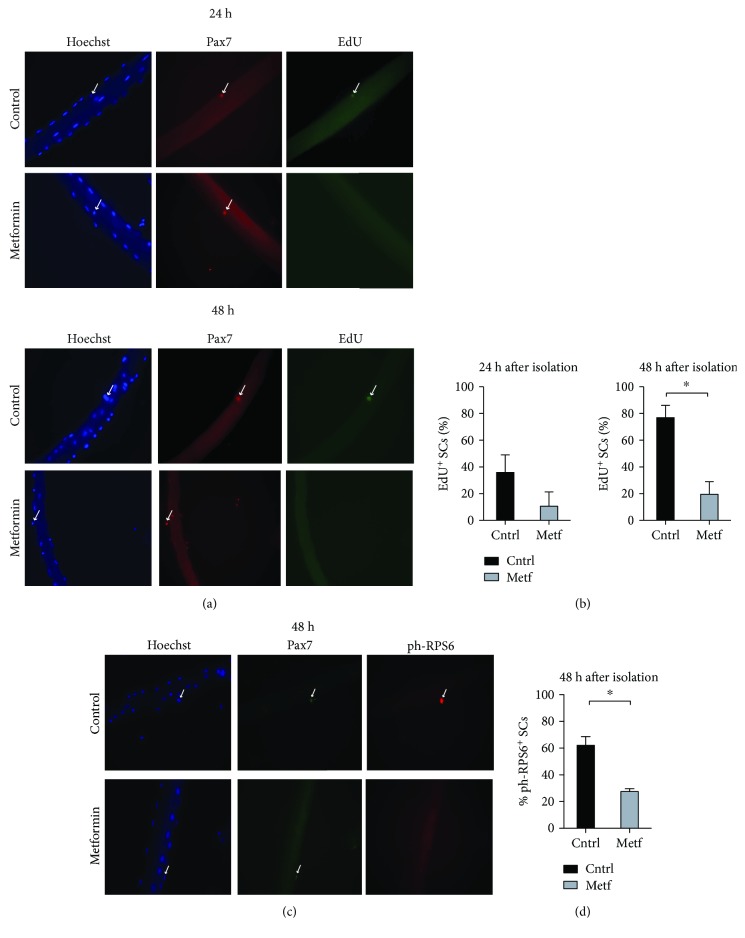
Metformin delays SC activation upon administration *in vivo*. (a) Single myofibers were isolated from control and metformin-treated C57BL/6 mice and cultured *in vitro* in Tyrode's medium containing an EdU labeling agent. The SCs associated with the myofibers were analyzed by immunofluorescence microscopy for the expression of Pax7 and the incorporation of EdU after 24 h and 48 h of culture. (b) Percentage of EdU-positive SCs associated with single myofibers isolated from control and metformin-treated C57BL/6 mice. The SCs associated with the myofibers were analyzed by immunofluorescence microscopy for the expression of Pax7 and the incorporation of EdU after 24 h and 48 h of culture (n24h cntrl = 48 SCs, n24h metf = 40 SCs, n48h cntrl = 40 SCs, and n48h metf = 36 SCs). (c) Single myofibers were isolated from control and metformin-treated C57BL/6 mice and cultured *in vitro*. The SCs associated with the myofibers were analyzed by immunofluorescence microscopy for the expression of Pax7 and ph-RPS6 after 48 h of culture. (d) Quantitation of the percentage of myofibers associated SCs positive for ph-RPS6 after 48 h in culture. Myofibers were isolated from control and metformin-conditioned C57BL/6 mice (n48h cntrl = 42 SCs, n48h metf = 40 SCs).

We further evaluated the perturbation of SC activation induced by in vivo metformin administration. C57BL/6 mice received metformin in water for 21 days, and individual myofibers were isolated and cultured *in vitro* for 24 h and 48 h. The propensity of SCs to proliferate was monitored by measuring EdU incorporation in Pax7^+^ cells while metabolic activation was assessed by looking at the phosphorylation of RPS6. In metformin-treated mice, 24 hours after myofiber isolation, fewer than 10% SCs are observed to incorporate EdU. After 48 hours, this percentage almost doubles but it is still significantly lower than the 80% EdU-positive SCs in the untreated controls (Figures 7(a) and 7(b)). Moreover, the SCs in myofibers from metformin-treated mice exhibit a significantly reduced phosphorylation of RPS6 (Figures 7(c) and 7(d)). In conclusion, exposure of SCs to metformin either *in vivo* or ex vivo delays their activation.

### 3.7. Sirt-1 Activity in SCs Is Not Affected by Metformin Treatment

**Figure 8:**
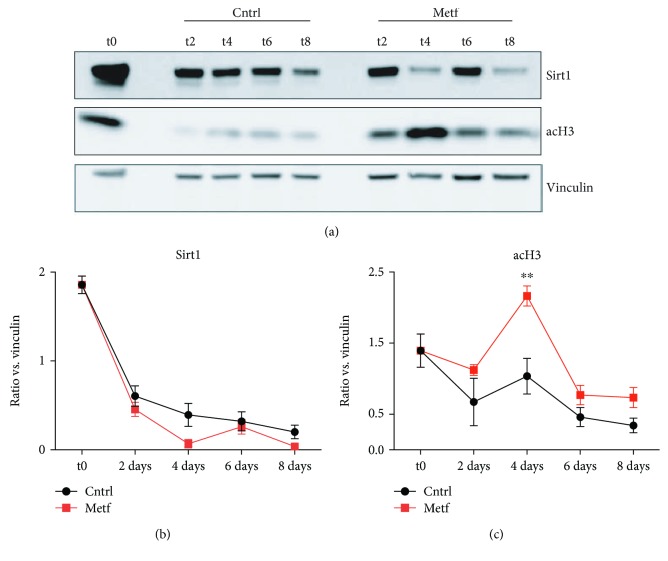
Sirt1 levels in metformin-treated SCs. (a) Western blot analysis for the expression of Sirt1 and acH3 in control and metformin-treated SCs after 2, 4, 6, and 8 days of treatment (t2, t4, t6, and t8, respectively). Vinculin was used as a loading control. (b) Quantitation graph of Sirt1 protein levels monitored by western blot in four independent cell isolations and biological replicates (*n* = 4). Statistical significance was evaluated by the ANOVA test (^∗^ *p* < 0.05). (c) Quantitation graph of acH3 protein levels monitored by western blot in four independent cell isolations and biological replicates (*n* = 4). Statistical significance was evaluated by the ANOVA test (^∗^ *p* < 0.05).

Given that metformin mimics glucose restriction and affects SC metabolism by activating AMPK and by inhibiting the downstream marker mTOR, we asked whether these effects are mediated by Sirt1 activation. To this end, we analyzed the protein levels of Sirt1 and acetylated H3 (acH3) by western blot in SCs treated with 2 mM metformin *in vitro* for 2, 4, 6, and 8 days (Figure 8(a)). As shown in Figure 8, metformin does not affect Sirt1 levels (Figure 8(b)) while acetylation of H3 is increased upon 4 days of metformin treatment (Figure 8(c)). From the above results, we can conclude that metformin's effect on SC activation and differentiation is not mediated by Sirt1 activation.

### 3.8. Metformin Induces a Mitochondrial Stress in SCs *In Vitro*

**Figure 9:**
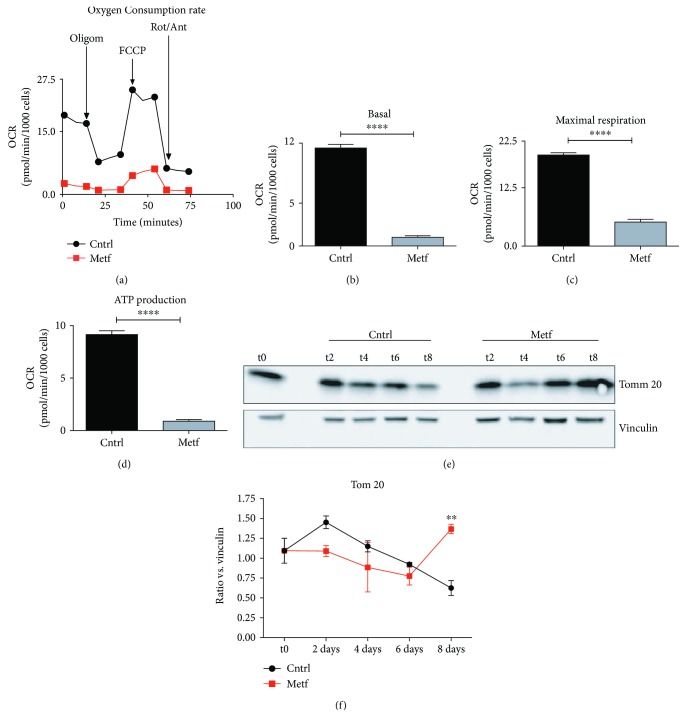
Metformin inhibits mitochondrial respiration in SCs. (a) Oxygen consumption rate (OCR) of the Mito Stress Test conducted with a Seahorse Extracellular Flux Analyzer. OCR is expressed in pmol of O_2_ consumed per minute, normalized over the number of cells in the assay well. Oligomycin (Oligom.), FCCP, and rotenone/antimycin (Rot/Ant) were sequentially added to the well to perturb the electron transport chain machinery. (b–d) Bar plot graphs of mitochondrial functional parameters: (b) basal respiration is the delta between the basal OCR and the lowest plateau reached upon Rot/Ant injection, (c) maximal respiration is inferred as the delta between Rot/Ant and FCCP maximal plateau, and (d) ATP production is referred to as the delta between the basal and the oligomycin injection. Statistical analysis was performed through Student's *t*-test (^∗∗∗∗^ *p* < 0.0001, *n* = 4). (e) Western blot analysis of Tom20 protein levels in control and metformin-treated SCs after 2, 4, 6, and 8 days of treatment (t2, t4, t6, and t8, respectively). Vinculin was used as a loading control. (f) Quantitation graph of Tom20 protein levels monitored by western blot in four independent cell isolations and biological replicates (*n* = 4). Statistical significance was evaluated by the ANOVA test (^∗∗^ *p* < 0.01).

Since metformin is known to target the mitochondrial respiratory complex, we next asked whether the observed effect of metformin on satellite cell differentiation was accompanied by a perturbation of metabolism. In order to assess the effect of metformin on SC mitochondrial function, we isolated SCs and treated them *in vitro* for 24 h with 2 mM metformin. Following treatment, oxygen consumption was measured with a Seahorse instrument after sequential drug treatment. As shown in Figure 9(a), metformin negatively affects the SC mitochondrial respiratory flux and decreases both basal respiration (Figure 9(b)) and maximal respiration (Figure 9(c)).

A decrease in basal respiration could be caused either by a reduction in the number of mitochondria or by a decline in the activity of the respiratory chain. To distinguish between the two alternatives, we measured the levels of the mitochondrial structural protein Tom20. As shown in Figure 9(f), the levels of Tom20 do not significantly decrease after metformin treatment, confirming that metformin affects the efficiency of the respiratory chain. On the contrary, at day 8, the protein levels of Tom20 significantly increase in the metformin-treated samples, which is in accord with the belated differentiation of metformin-treated SCs since it is known that myogenic differentiation is followed by a switch to oxidative phosphorylation metabolism [29, 30].

### 3.9. Metformin-Treated SCs Are Transcriptionally Quieter and Smaller in Size

**Figure 10:**
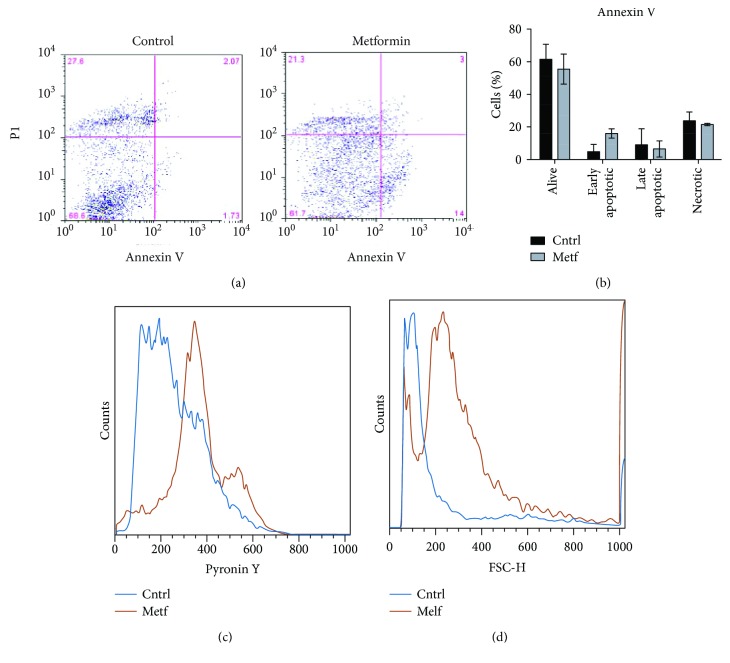
*In vitro* metformin-treated SCs retain stem cell characteristics. (a) Dot plot of flow cytometric analysis of apoptotic cells after metformin treatment *in vitro*. Cell populations: alive cells (annexin V negative, PI negative), early apoptotic cells (annexin V positive, PI negative), late apoptotic cells (annexin V positive, PI positive), and necrotic cells (annexin V negative, PI positive). The position of the quadrant lines was stabilized based on the distinguishing cell populations. (b) Quantitation of the percentages of alive, early apoptotic, late apoptotic, and necrotic cells (n experimental replicates = 2). (c) Representative histogram of the forward scattering (FSC) parameter by FACS analysis in control and metformin-treated SCs. (d) Representative histogram of pyronin Y FACS analysis in control and metformin-treated SCs.

We finally asked whether metformin treatment affects the proliferation potential and metabolic activation of isolated SCs. SCs, isolated from C57BL/6 mice and treated *in vitro* with 2 mM metformin immediately after isolation, fail to attach to the gelatin-coated culture dish even after 4 days, whereas most control SCs adhere to the plastic dish and start proliferating (Figure S3). Analysis of apoptosis with annexin V/propidium iodide (PI) double staining revealed a small, nonsignificant increase of early apoptotic cells in these SCs treated with metformin (Figures 10(a) and 10(b)). In addition, SCs treated with metformin immediately upon isolation for 48 h are smaller in size compared to the untreated control (Figure 10(c)) and display reduced levels of pyronin Y staining (Figure 10(d)). Pyronin Y is an RNA intercalator and is used here as a measure of RNA content. This staining protocol allows distinguishing quiescent cells (with low RNA levels) from activated cells in G1 which are characterized by higher RNA levels [31].

The above results show that metformin-treated SCs are small quiescent cells characterized by a reduced transcriptional activity and a limited potential to grow as adherent cells compared to the untreated counterparts. These observations are consistent with a model implying a delay in SC exit from quiescence following treatment with metformin.

## 4. Discussion

In the unperturbed adult muscle, SCs are mitotically quiescent. Maintenance of quiescence and the ability to regain quiescence after activation are essential for the long-term homeostasis of the stem cell pool [32, 33]. The balance between cell quiescence, differentiation, and renewal is one of the most important factors for efficient regeneration after tissue damage and aging or in disease.

Aged SCs fail to maintain quiescence, and once activated, commitment to the myogenic lineage is favored at the expense of self-renewal [34]. Along the same lines, one of the pathological features of Duchenne muscular dystrophy is the depletion of the SC pool induced by repeated cycles of degeneration-regeneration [35–37]. Understanding and learning to control the mechanisms involved in the establishment and maintenance of SC quiescence remain an issue of interest. The SC state is influenced by a variety of intrinsic and extrinsic factors, with the microenvironment and the stem cell niche having unique and indispensable roles.

Recent studies have highlighted the role of metabolism in SC activation and function. SC number, differentiation potential, and functional engraftment efficiency are increased following calorie restriction in either the donor or the recipient mice [13]. Furthermore, SCs derived from mTOR-knockout mice exhibit defective proliferation and differentiation kinetics and express lower levels of Pax7, Myf5, MyoD, and myogenin [38]. Recently, Haller et al. have reported the transient activation of mTOR signaling in different cell types during tissue regeneration [39]. However, after repeated regeneration cycles, mTOR signaling has an inhibitory role on stem cell maintenance. It was proposed that pharmacological inhibition of mTOR by rapamycin is sufficient for stem cell pool maintenance in different tissues, including muscle stem cells.

Building on these studies, we investigated the effect of metformin, a calorie restriction mimicking drug that inhibits the mTOR pathway, on the activation, proliferation, and differentiation of SCs. In the previous study, we have demonstrated that metformin inhibits C2C12 myogenic differentiation, by preventing irreversible cell cycle exit and reducing MyoD and p21cip1 levels [12]. Our current study extends the effect of the drug on primary SCs *in vitro*, ex vivo, and *in vivo*. In particular, our results show that isolated SCs, treated *in vitro* with 2 mM metformin, retain the expression of Pax7 for a longer time compared to controls. The expression of Pax7 is accompanied by a prolonged phase of BrdU incorporation, which indicates that metformin belates the final differentiation.

The delayed Pax7 downregulation is accompanied by a belated expression of myogenic differentiation markers (myogenin and MyHC) and by a reduced fusion index. However, myogenin levels were significantly lower only at day 2 in the metformin-treated samples.

Since Pax7 promotes progenitor commitment to the muscle lineage, we hypothesized that metformin delays the process of SC progenitor activation and entry into the cell cycle. In order to gain further insight into the molecular underpinnings of the role of metformin in SC activation, we isolated individual myofibers from C57BL/6 mice and treated them *ex vivo* with 2 mM metformin. The SCs associated with metformin-treated myofibers exhibit reduced EdU incorporation after 24 h and 48 h of culture when compared to controls. This low propensity to proliferate is matched by a decreased ribosome protein S6 (RPS6) phosphorylation. Likewise, the reduction in the level of phospho-RPS6 is observed also in isolated SCs that were cultured and treated with 2 mM metformin for 4 days *in vitro*. RPS6 is a downstream target of mTOR, which is inhibited by the metformin-activated AMPK. Thus, metformin hinders translation by inhibiting the mTOR/P70S6K, RPS6, and 4E-BP1 axis.

We extended these observations *in vivo*, by studying the regeneration of skeletal muscle and the activation of SCs upon muscle injury. After metformin administration, mice were subjected to muscle damage by CTX and the regeneration process was monitored by measuring the number of centronucleated myofibers and myofiber cross-sectional area (CSA) at 4 and 7 days of postinjury. The percentage of centronucleated myofibers in the metformin-treated mice is lower than that in the control at 4 days after injury. Moreover, at 7 days of postinjury, control myofibers show a larger cross-sectional area (500 ha^2^) than the metformin-conditioned mice that still retain more myofibers with a CSA around 250 co^2^. These data suggest that metformin-treated mice are still fully regenerating 7 days after injury. Furthermore, SCs isolated from metformin-conditioned mice differentiate and fuse into myotubes later compared to controls *in vitro*. This is consistent with the hypothesis that metformin delays skeletal muscle regeneration *in vivo*.

We additionally isolated individual myofibers from C57BL/6 mice treated with metformin for 21 days and cultured them *in vitro*, in EdU-containing medium. We observed a reduced percentage of proliferating SCs (i.e., positive for EdU). A similar result was also obtained when myofibers were treated with metformin *in vitro*. Notably, this nonproliferating phenotype is accompanied by a reduction in the percentage of ph-RPS6-positive SCs.

To further gain an insight into the mechanisms by which metformin disturbs SC metabolism, resulting in their activation and proliferation, we focused on the expression of Sirt1. Sirt1 is one of the most important class III histone deacetylases that has been proposed to play a crucial role as a metabolic regulator in different cell types. Specifically in muscle, Ryall et al. have demonstrated that upon activation SC switch to glycolysis, intracellular NAD^+^ levels are decreased and subsequently the activity of Sirt1 is inhibited [40]. As a result, H4K16 acetylation increases and the transcription of the myogenic genes begins. On the other hand, Tang and Rando proposed that SC exit from quiescence necessitates an increase in autophagic flux which is mediated by Sirt1 [10]. In our conditions, we observed that Sirt1 levels remain unaffected upon metformin treatment and this result allows us to conclude that metformin's effects on SCs are not mediated by Sirt1.

On the other hand, after analysis of the SC mitochondrial respiration, we noted a significant impairment of oxidative phosphorylation in the metformin-treated samples. By further analyzing the levels of Tom20 protein, a mitochondrial structure marker, we observed that metformin does not significantly impact Tom20 levels, suggesting that the drug affects the efficiency of the respiratory chain. However, Tom20 levels significantly increase at day 8 in the metformin-treated SCs, which is in accordance with the fact that metformin delays SC differentiation.

Ultralow attachment culture dishes allow formation of myospheres [41] from muscle-derived cells by providing a niche-like environment and by helping maintain a more primitive cell state [42]. Given that metformin delays the activation of SCs associated with individual myofibers, we extended our observations by treating SCs with metformin immediately after isolation in order to check their stem cell characteristics. Interestingly, different from control cells, metformin-treated cells do not attach to the gelatin-coated cultured dishes and remain alive in suspension. The nonadherent metformin-treated SCs do not show significant signs of apoptosis and appear to be smaller and transcriptionally quieter than the control.

Our results suggest that metformin delays SC activation by preserving them in a less active metabolic state. As a result, metformin-treated SCs are delayed in their differentiation *in vitro.* Consistently, the process of muscle regeneration after cardiotoxin injury *in vivo* is also delayed.

The importance of nutrient availability and metabolism in the process of differentiation has been highlighted by different studies. Our data are in accordance with the work of Rodgers et al. [11] which reports that mTOR activity is necessary and sufficient for the transition of SCs from a quiescent G_0_ phase into a quiescent, more stress responsive, G_Alert_ phase. This intermediate G_Alert_ state allows SCs to perform their first division faster than SCs in the G_0_ state. G_Alert_ is associated with activated mTOR and RPS6 signaling, which are negatively modulated by metformin. In addition, our results strengthen the notion that quiescent SCs have a low metabolic rate and support the view that their activation and entry into the cell cycle can be manipulated by interfering with their metabolism [43]. Finally, our results are in accord with mTOR being responsible for the activation of different somatic stem cell populations and support the exploration of its pharmacological perturbation as a useful tool for stem cell maintenance [39].

One of the current goals in the field of skeletal muscle biology is to understand the etiology of SC exhaustion during aging and improve muscle function by targeting the rejuvenation of the old SC population. Both intrinsic and extrinsic factors of the SC environment seem to be involved in aged SC loss of function, such as increased DNA damage, epigenetic modifications, or altered metabolic signaling [44]. In 2013, Sandri et al. [45] demonstrated an increased phosphorylation of RPS6 and activation of mTOR signaling in aged mice, in accord with the findings of Cerletti et al. [13] that AMPK activation by a low-calorie diet improves the activity of SCs in the muscles of old mice. Other studies have focused on the pharmacological inhibition of p38 MAPK [46], silencing of the p16 cell cycle regulator [47], or augmentation of the autophagic flux [48] as different approaches for the rejuvenation of the geriatric muscle. In this context, our study suggests that the metabolic perturbation induced by metformin, by forcing SCs into a low metabolic state, sustains their persistence in a quiescent state. These findings highlight a possible use of metformin as a pharmacological intervention for muscle stem cell maintenance during the repeated regeneration cycles in disease [49, 50] or in aging, when chronic activation of mTOR signaling occurs [51, 52].

## Data Availability

The data used to support the findings of this study are available from the corresponding author upon request.

## Supplementary Materials

Supplementary 1 Figure S1: Pax7 and MyoD protein levels, monitored by western blot, are not affected by metformin. (a) SCs treated upon attachment with 2 mM metformin were analyzed for the expression of Pax7 and MyoD by western blot after 2, 4, 6, and 8 days of treatment (t2, t4, t6, and t8, respectively). Vinculin was used as a loading control. (b) Quantitation graphs of Pax7 and MyoD protein levels monitored by western blot in four independent cell isolations and biological replicates (*n* = 4). Statistical significance was evaluated by the ANOVA test (^∗^ *p* < 0.05).

Supplementary 2 Figure S2: metformin delays SC differentiation in mice after CTX muscle injury. (a) SCs isolated from control and metformin-treated mice 1, 2, 4, and 7 days after CTX treatment were cultured *in vitro* and examined for their myogenic differentiation potential 2 days after attachment by immunofluorescence analysis. The expression of MyHC was used as a differentiation marker. (b) The fusion index was calculated as the % ratio of the nuclei inside the myotubes to the total number of *nuclei*. Statistical significance was evaluated by Student's *t*-test (^∗^ *p* < 0.05).

Supplementary 3 Figure S3: metformin-treated SCs do not attach on gelatin-coated plates. (a) Representative bright-field images of SCs treated with 2 mM metformin immediately after isolation. SCs were isolated from C57BL/6 mice and incubated on a gelatin-coated culture dish for 4 days.
